# Understanding the family burden and caregiver role in stroke rehabilitation: insights from a retrospective study

**DOI:** 10.1007/s10072-024-07668-5

**Published:** 2024-07-03

**Authors:** Maria Grazia Maggio, Francesco Corallo, Morena De Francesco, Maria Cristina De Cola, Rosaria De Luca, Alfredo Manuli, Angelo Quartarone, Amelia Rizzo, Rocco Salvatore Calabrò

**Affiliations:** 1 IRCCS Centro Neurolesi Bonino Pulejo, S.S. 113 Contrada Casazza, Messina, 98124 Italy; 2 University of Messina, Piazza Pugliatti, 1, Messina, 98120 Italy; 3 Physical Medicine and Rehabilitation Unit, Piazza Pugliatti, 1, Messina, 98120 Italy

**Keywords:** Burden, Caregiver, Quality of Life, Rehabilitation, Stroke

## Abstract

**Introduction:**

Stroke negatively impacts both patients and their families, who must face multiple changes after the onset of the disease. Family caregivers must face new problems with a possible sense of inadequacy, stress and burden. Our retrospective study aimed to assess the burden of caregivers during the rehabilitation process of patients with Stroke.

**Material and method:**

This study included patients with a diagnosis of stroke and their caregiver, who attended the Day Hospital of the IRCCS Neurolesi Center "Bonino-Pulejo", Messina, Italy, between January 2018 and October 2019, using electronic recovery system data. The final sample consisted of 30 patients and their caregivers.

**Results:**

Significant improvements were observed in patients' cognitive and mood scores, reflecting the efficacy of rehabilitation therapies. Additionally, a correlation emerged between patients' reported anxiety levels and caregivers' reported depression levels, highlighting a dynamic interaction between the emotional states of the two groups.

**Conclusion:**

The study highlights the intricate interplay between caregiver characteristics, patient outcomes, and family dynamics in the context of caregiving. Targeted interventions aimed at improving family resilience and coping mechanisms are crucial to optimizing the well-being of both caregivers and patients.

## Introduction

Due to its epidemiological characteristics, stroke represents a serious global public health problem and a major cause of disability and death. Overall, in Italy every year, approximately 185,000 people are affected by cerebral stroke [1]. The incidence is proportional to the age of the population, gradually increasing and rising to 70–75% over 65 years of age; 10–20% of people affected by stroke for the first time die within a month and another 10% within the first year [1–3]. The pathology causes multiple disabilities, and early recovery is crucial, especially during the first 3–6 months to improve patients' physical, emotional, and cognitive abilities in daily activities, prevent complications, and reduce disability [4]. Stroke negatively impacts both patients and their families, who must face multiple changes after the onset of the disease. In fact, the sudden onset and symptoms following stroke can have a long-lasting impact on patients and their family caregivers, both on an individual and relational level [5]. Patients and caregivers can experience negative psychosocial consequences (anxiety, depression, social problems, isolation) and a reduced quality of life [6–8], involving negative emotions and lifestyle changes. Indeed, stroke can be a challenge with significant effects on family, such as on communication, relationships, resilience, and family functioning [8–10]. Family caregivers can offer important support to stroke patients and take on this role immediately after the stroke event [11]. A family caregiver is a family member, such as a spouse, child, friend, or unpaid neighbor, who provides care to a person with a chronic illness who needs assistance with daily living tasks, such as bathing, dressing, and taking medicine [12]. However, family caregivers often must suddenly assume their new role, resulting in changes in the family functioning pattern [13–16]. During this period, a family caregiver must face new problems with a possible sense of inadequacy due to a lack of knowledge and skills necessary to carry out the role of care and assistance, such as managing medications, preparing food, and supporting the patient [17]. The intense stress caused by the hard work of care and assistance over a long period of time has been defined as a "family burden" [6–8]. The latter is due to the objective and subjective consequences linked to caring for a family member suffering from a serious disorder. In fact, caregivers can have a high economic and psychological burden, as well as a possible loss of psycho-physical well-being [18–23]. In this context, other close family members can support the primary caregiver through direct or indirect supervision, help and assistance in carrying out paperwork and medical visits; assistance with housework or travel [24]. Therefore, the ictal event may impose a reorganization of the family that affects all members of their family. According to various authors [13–16], family functioning is influenced by the members' phases of re-elaboration: initially, they experience "shock" (despair, anguish) and denial (rejection and search for some type of magic solution); followed by reorganization and eventual acceptance, which leads to a strengthening of the new equilibrium. Consequently, the family is to be considered in the rehabilitation process as a fundamental ally and resource [16]. In agreement with Rolland [13–15], when taking care of a patient with a stroke it is essential to adopt a systemic approach that considers not only the patient but also the family. In a previous study, we observed that patients with Acquired Brain Injury could exhibit enhanced compliance and treatment response when their family actively participates in their management and care [10].

From this perspective, our retrospective study aimed to assess the burden of caregivers during the rehabilitation process of patients with stroke.

## Materials and methods

### Study design and population

This study included patients with a diagnosis of stroke and their caregiver, who attended the Day Hospital of the IRCCS Neurolesi Center "Bonino-Pulejo", Messina, Italy, between January 2018 and October 2019, using electronic recovery system data. The study's retrospective nature and the extraction from an electronic medical record have minimized the scoring bias. This retrospective study was conducted following the 1964 Helsinki Declaration and was approved by local ethical committee. Before entering the study, written informed consent was obtained from all subjects.

Initially, we included 45 patients who encountered the study criteria: fifteen of these (33.3%) were excluded because they did not complete the rehabilitation process, probably due to the fatigue experienced during the rehabilitation sessions with the robotic devices, or due to the lack of tests completed by the caregivers. The final sample consisted of 30 patients and their caregivers. We selected patients with stroke in the post-acute phase (i.e. 3—6 months from the acute neurological event), and the paired caregivers. The data was collected retrospectively and then analyzed. The patients and the paired caregiver had signed a general informed consent on the use of the data for research purposes at the beginning of hospital admission. To be included in the study, caregivers had to be at least 18 years of age, and have no severe cognitive deficits or medical illness. The primary caregiver has been defined as the person who lives with the patient in the same home and takes primary responsibility for providing care to the patient at home.

Inclusion criteria for the patients were: i) neurological diagnosis of stroke (either ischemic or hemorrhagic);ii) in the chronic phase (at least 6 months from the acute event); (iii) age between 18 and 65; iiv) the presence of a family member who had completed the caregiver burden and mood tests.

### Procedures

Upon admission, all patients were assessed via motor and cognitive screening tests to determine the most suitable treatment path, which could include robotic rehabilitation. The treatment plan included a combination of traditional physiotherapy, speech therapy, and psychological support, integrated with physical therapy using innovative technologies such as focal vibrations, robotic devices, and advanced cognitive rehabilitation techniques, including virtual reality (for more detail see 25,26). Our advanced devices allow to target several areas, including enhancing upper and lower extremity strength, improving balance and muscle tone, and optimizing cognitive functions through the combined use of tools such as virtual reality [25, 26]. We have developed specific technologies to optimize the efficiency and repeatability of movements, thus reducing the time and effort required for rehabilitation and increasing both the intensity and volume of motor exercises [25, 26]. The concomitant use of robotics and virtual reality not only stimulates the patient's motivation and active participation but also improves the overall effectiveness of the treatment, offering a range of engaging activities. This personalized approach improves patient compliance with the rehabilitation program [26]. Furthermore, during the evaluation process, the emotional state of the caregivers towards the patient is also taken into consideration in providing them with adequate support [11].

### Outcomes measures

Based on the study objectives, we selected patients who had been evaluated using the Montreal Cognitive Assessment (MoCA) test, which evaluates global cognitive function; the Hamilton Anxiety Rating Scale and the Hamilton Depression Rating Scale, which allows the assessment of anxiety and depression levels. Caregivers were indeed recruited based on whether they were tested with the Hamilton Anxiety Rating Scale and the Hamilton Depression Rating Scale, along with the Caregiver Burden Inventory (CBI) [27, 28], which allows assessment of their emotional state and the impact of caregiving responsibilities on their well-being. Because this was a retrospective study, only patients who had undergone these specific assessments and their caregivers were included in the analysis.

### Statistical analysis

Data were analyzed using the R software—version 4.3.0, considering p-value < 0.05 as statistically significant. Since the not normal distribution for most of the target variables, measured by the Shapiro–Wilk statistic, non-parametric analysis was performed. Thus, the Wilcoxon signed-rank test was used to compare patients’ clinical assessment between baseline and the end of the study. The Spearman's rank correlation coefficient was used to measure correlations between caregivers’ test scores and patients’ score differences between assessment times, i.e. T1-T0 score differences.

We subdivided patients into two groups according to the caregivers' CBI score, choosing 24 as the cut-off of the CBI score [27], and compared patients’ clinical scores between these two groups by means of the Mann–Whitney U test. In addition, we used the car package of R to carry out an analysis of covariance (ANCOVA) for any patient’s outcome measure. The models had the test score at T1 as dependent variable, the categorical variable ‘Group’ (low = CBI < 24; high = CBI ≥ 24) as independent variable, and the outcome score at baseline (T0) as covariate. ANOVA was used to verify whether the interaction term effect “outcome score at baseline * categorical variable” significantly affected the ANCOVA model. Both the assumption of homogeneity of regression slopes, as well as the homogeneity of variance assumption were assessed by ANOVA and the Levene’s test, respectively. The F-statistic and the adjusted R^2^ of the ANCOVA model were used as standardized measure of effect sizes. We repeated the same analysis subdividing patients into two groups according to the family relationship with caregivers (1 = partner; 0 = son/daughter).

## Results

Thirty patients with Stroke (mean ± SD age: 57.07 ± 11.80 years; 66.7% males) and their caregivers (mean ± SD age: 52.23 ± 13.80 years; 43.3% males) were enrolled in this study. A more detailed description of the two groups is in Table 1.

**Table 1:** Demographic characteristics of the sample

	Patients	Caregivers
Subjects	30	30
Age (years)	57.1 ± 11.8	52.2 ± 13.8
Female	10 (33.3%)	17 (56.7%)
Education (years)	11.7 ± 3.4	-
Family relationship		
Partner	-	23 (76.7%)
Son/Daughter		7 (23.3%)

Mean ± standard deviation was used to describe continuous variables; proportions (numbers and percentages) were used to describe categorical variables

As shown in Table 2, at the end of the rehabilitation treatment patients had significant T1-T0 differences in all test scores. We found a moderate correlation between the patients’ HRS.A score T1-T0 difference and the caregivers’ HRS.D scores (r = -0.36), as well as between the patients’ HRS.D score T1-T0 difference and the caregivers’ CBI.EM scores (r = 0.31).

**Table 2:** Statistical comparisons of patients’ clinical scores between baseline (T0) and follow-up (T1) and caregivers’ clinical scores

Clinical assessment	Patients		P	Caregivers
	T0	T1		T0
MoCA	23.5 (19.25–26.0)	25.0 (21.25–28.0)	0.002**	-
HRS-A	11.0 (4.0–17.5)	6.5 (4.0–12.25)	0.025*	11.0 (4.0–15.0)
HRS-D	12.5 (6.25–14.75)	7.0 (5.0–11.5)	0.004**	11.0 (8.0–13.75)
CBI	-	-	-	20.5 (15.0–35.75)

Scores are in median (first-third quartile); significant differences are * < 0.05; ** < 0.01; *** < 0.001

Montreal Cognitive Assessment (MoCA); Hamilton Rating Scale—Anxiety (HRS-A); Hamilton Rating Scale—Depression (HRS-D); Caregiver Burden Inventory (CBI)

When we divided the patients according to the caregivers’ CBI cut-off value, we found at baseline a significant difference in patients’ HRS-A scores (p = 0.034). On the contrary, no significant differences at follow-up emerged, as viewable in Table 3.

**Table 3:** Statistical comparisons of patients’ clinical scores between baseline (T0) and follow-up (T1). Patients were divided into two groups based on the caregivers' CBI score or according to the family relationship with caregivers

Clinical assessment	Baseline		*p*-value	Follow up		*p*-value
	Caregivers CBI < 24	Caregivers CBI ≥ 24		Caregivers CBI < 24	Caregivers CBI ≥ 24	
MoCA	23.5 (20.5–25.7)	23.5 (18.5–26.5)	0.534	25.0 (21.0–28.0)	25.0 (22.7–28.0)	0.457
HRS-A	8.0 (3.0–14.7)	14.0 (11.5–20.5)	0.034*	6.0 (3.2–8.0)	11.0 (4.0–14.5)	0.255
HRS-D	12.5 (7.2–14.0)	7.0 (5.2–9.0)	0.383	12.5 (6.5–16.2)	9.5 (3.0–12.2)	0.069
	Partner	Son/Daughter		Partner	Son/Daughter	
MoCA	24.0 (21.0–26.0)	19.0 (14.5–27.0)	0.216	25.0 (22.5–28.0)	21.0 (17.5–27.5)	0.133
HRS-A	10.0 (4.0–18.0)	12.0 (6.0–16.0)	0.662	7.0 (3.5–11.5)	6.0 (4.5–10.5)	0.222
HRS-D	12.0 (6.5–14.5)	13.0 (8.0–16.5)	0.422	8.0 (5.5–12.0)	7.0 (4.0–8.0)	0.451

Significant differences are * < 0.05; ** < 0.01; *** < 0.001

Montreal Cognitive Assessment (MoCA); Hamilton Rating Scale—Anxiety (HRS-A); Hamilton Rating Scale—Depression (HRS-D)

Assumptions of homogeneity of regression slopes and covariate-treatment independence were tenable in all covariate models, except in HRS-D (F (1) = 5.6; p = 0.02). The interaction term was not considered in the ANCOVA models fitting, because ANOVA has shown that this term did not bring significant information to the covariate models. ANCOVA results showed that caregiver burden did not affect patient recovery, as reported in Table 4.

**Table 4:** ANCOVA results for each covariance model. Patients were divided into two groups based on the caregivers' CBI score or according to the family relationship with caregivers

Groups	Clinical assessment	Group coefficient				Adjusted R^2^
		Estimate	Std. Error	t value	p value	
CBI	MoCA	0.25	0.70	0.35	0.73	0.64
	HRS-A	0.83	1.51	0.55	0.59	0.45
	HRS-D	Not Applicable				
Family relationship	MoCA	-0.93	0.81	-1.14	0.26	0.66
	HRS-A	-1.03	1.24	-0.83	0.42	0.22
	HRS-D	-0.67	1.65	-0.41	0.69	0.44

Significant differences are * < 0.05; ** < 0.01; *** < 0.001

Montreal Cognitive Assessment (MoCA); Hamilton Rating Scale—Anxiety (HRS-A); Hamilton Rating Scale—Depression (HRS-D)

When we divided the patients according to the family relationship with caregivers, no significant differences in all outcome scores emerged (Table 3). Assumptions of homogeneity of regression slopes and covariate-treatment independence were tenable in all covariate models, and the interaction term was not significant for any models. ANCOVA results show that caregiver relationship did not affect patient recovery (Table 4).

## Discussion

The study results highlight significant improvements in cognitive and mood scores (MOCA, HRS-A, HRS-D) from baseline (T0) to follow-up (T1), reflecting the positive impact of rehabilitation interventions on patients. Furthermore, a noteworthy correlation was found between patient-reported levels of anxiety and caregiver-reported levels of depression. This suggests a relationship between the emotional states of caregivers and patients. In line with our results, some authors have highlighted the existence of a dynamic interaction between the emotional states of caregivers and patients [29–31]. Bakas and colleagues [32] demonstrated how the mental health of stroke patients and their caregivers influence each other. A partner's anxiety and depression affect the patient, and vice versa, causing an increased risk of psychological problems. The existence of a significant relationship between patients' depression and their caregivers' depression was also demonstrated by Kotila and colleagues [33]. In agreement with these findings, other authors have highlighted the correlation between the emotional states of the patients and their caregivers [34–36]. Loh and colleagues [34] showed how caregivers present evident symptoms of anxiety and depression, associated with the disability of stroke patients, which can limit their role. Furthermore, it has been highlighted that the emotional state of caregivers can worsen patients' depressive symptoms and negatively influence rehabilitation. This is also demonstrated by the studies of Choi-Kwon and colleagues which highlight how anxiety and depression of both caregivers and patients are important variables that influence the overall burden of the caregiver [37].

In this sense, significant disparities in anxiety scores were observed among patients based on the level of burden on their caregivers. Caregivers who perceived a high level of burden tended to have patients with high levels of anxiety. Instead, caregivers subjected to a lower care burden were linked to patients with lower levels of anxiety, highlighting the profound influence of the caregiver on the patient's well-being [38].

However, other authors [39] found that the caregivers' psychological well-being could be strictly associated to a better level of caregiving and to a better functional outcome of the patients that, in turn, could positively influence the caregivers' psychological well-being. Another study found that during the COVID pandemic, the physical absence of caregivers in the neurorehabilitative played a role in hindering the functional outcome of the patients, thus further showing the virtuous circle between an effective style of caregiving and the outcome of the loved ones [40].

Our study is in line with the results of various authors [10, 39, 41], who highlight how the perception of care burden tends to change depending on the type of relationship with the patient. Some authors define burden as the impact on the family determined by changes in the patient's well-being [19–22]. The burden is a multidimensional variable [19, 20] that can lead the caregiver to underestimate or delay their needs, causing negative experiences, such as the reduction of interpersonal relationships and a worsening of the quality of life [21, 22]. The caregiver's well-being is an important factor that constantly changes based on the satisfaction of her needs, environmental conditions, and the workload perceived by the caregiver. In turn, this well-being affects the way the patient is cared for, as observed by several authors [10, 21, 22, 39, 40]. The objective burden (burden) and worry (tension) for patients can have repercussions on the quality of the care itself. It has been reported that caregivers with a good level of well-being provide better care to their relatives and better cope with negative conditions resulting from the disease [23]. Moreover, other research suggests that caregivers often report altered relationships with the survivor [42] and within the family [43, 44] following the illness, impacting their own and the patient's well-being, as observed in our sample.

Another interesting finding was that our sample showed variations in anxiety and depression scores across different types of caregivers, with children serving as caregivers reporting higher levels of both anxiety and depression than spousal caregivers [45–47]. This highlights the complex dynamics within family systems and the unique challenges faced by children in transitioning into caregiving roles for their parents, particularly in the context of post-trauma family role reorganization. According to these results, Bastawrous et al. highlighted that changes in the parent–child relationship contribute to feelings of sadness, loss, and frustration [48]. For example, many daughters have difficulty providing intimate care (e.g., toileting and bathing) [48]. Furthermore, caregivers of children identified changes in their relationship with parents following the stroke, including a “role reversal” as they now cared for those who once cared for them [49]. Furthermore, children feel a “moral duty” to care for those who care for them [48]. Other studies have reported that due to changes in typical marriage [48, 49] and parent–child roles [48], both spouse and child caregivers experienced a sense of loss of relationship with the surviving partner [48–50]. For spouses, there is a sense of loss of the partner, and a reduction in intimacy is due to the change in the role of caretaker, which translates into the partner's role as "a stranger to being cared for". This sense of loss is different in children, as the loss of the role of the child is experienced more than the loss of the "parent" person, who however does not become a stranger to look after, resulting in a permanent depressive state [48, 50–53].

In conclusion, the study highlights the intricate interplay between caregiver characteristics, patient outcomes, and family dynamics in the context of caregiving. Targeted interventions aimed at improving family resilience and coping mechanisms are crucial to optimizing the well-being of both caregivers and patients [54–56]. Further research is needed that explores the long-term effects of caregiving interventions and their impact on family systems, drawing on systems theories to inform comprehensive and tailored support strategies.

## Data Availability

The data that support the findings of this study are not openly available due to reasons of sensitivity and are available from the corresponding author upon reasonable request.
